# Detection of *Aelurostrongylus* spp. Infection in the Neozoan American Mink (*Neogale vison*) in Southern Chile

**DOI:** 10.3390/pathogens15070757

**Published:** 2026-07-20

**Authors:** Joaquín Saavedra, Marcelo Gómez, Pamela Muñoz, Valentina Bernal, Pedro Aburto, Eduardo Raffo, Brandon Aristizabal, Manuel Moroni, Gisella Paredes, Andrea Monterroza, Rodrigo Arancibia, Anja Taubert, Carlos Hermosilla

**Affiliations:** 1 Facultad de Ciencias Veterinarias, Instituto de Farmacología y Morfofisiología, Universidad Austral de Chile, Valdivia 5090000, Chile; joaquin.saavedra@alumnos.uach.cl (J.S.); valentina.bernal@alumnos.uach.cl (V.B.); pedroaburto@uach.cl (P.A.); andremon9321@gmail.com (A.M.); rodrigo.arancibia@alumnos.uach.cl (R.A.); 2 Facultad de Ciencias Veterinarias, Instituto de Patología Animal, Universidad Austral de Chile, Valdivia 5090000, Chile; pamela.munoz@uach.cl (P.M.); brandon.aristizabal@alumnos.uach.cl (B.A.); manuelmoroni@uach.cl (M.M.); 3 Servicio Agrícola y Ganadero (SAG), Protección de Recursos Naturales Renovables, Valdivia 5090000, Chile; eduardo.raffo@sag.gob.cl (E.R.); gisella.paredes@sag.gob.cl (G.P.); 4 Biomedical Research Center Seltersberg (BFS), Institute of Parasitology, Justus Liebig University Giessen, 35392 Giessen, Germany; anja.taubert@vetmed.uni-giessen.de (A.T.); carlos.r.hermosilla@vetmed.uni-giessen.de (C.H.)

**Keywords:** American mink, nematode, parasites, metastrongyloid, *Aelurostrongylus*

## Abstract

Background: The American mink (*Neogale vison*) is an invasive mustelid in Chile that serves as a natural reservoir host for several pathogens. Cardiorespiratory nematodes of the superfamily Metastrongyloidea, including *Aelurostrongylus* spp., have been reported in carnivores worldwide but remain poorly documented in South American wildlife and invasive species. This study reports the first detection of *Aelurostrongylus* spp. infection in American minks in Southern Chile. Methods: Between 2024 and 2025, 27 American mink carcasses were collected from the Los Ríos and Los Lagos regions, Southern Chile. Complete necropsies were performed, and cardiorespiratory organs were examined for presence of parasites. Nematode larvae identified in bronchoalveolar lavage were characterized both morphologically and molecularly through PCR amplification, confirming their classification within the superfamily Metastrongyloidea. Results: Metastrongyloid nematodes were detected in 33.3% (9/27) of examined mink. Histopathological examination of lung tissue in some specimens identified adult parasite specimens. Morphological examination revealed *Aelurostrongylus* spp. first-stage larvae (L1) with characteristic morphology and morphometry. Molecular analysis confirmed the presence of *Aelurostrongylus* spp., with sequences showing 99% identity to *Aelurostrongylus abstrusus*. Most of the infected individuals were adults collected from freshwater environments. Conclusions: This study provides the first evidence of patent *Aelurostrongylus* spp. infection in neozoan American minks in South America, highlighting the role of this invasive species as a potential reservoir for cardiorespiratory parasites and emphasizing the need for continued surveillance of parasitic infections originating from invasive carnivores.

## 1. Introduction

The American mink (*Neogale vison*, formerly *Neovison vison*) is a semiaquatic mustelid native to North America that has become a neozoan species in several regions worldwide, including Chile [1,2]. Introduced to Chile in the 1930s for fur farming purposes, the American mink has since established feral populations that have expanded throughout southern regions of the country [3,4,5]. The current geographical distribution of American mink in Chile extends from Patagonia to the Araucanía region, where it occupies diverse freshwater and coastal ecosystems [4,5]. As an opportunistic meso-carnivorous predator, the American mink consumes a wide spectrum of prey, including endemic aquatic species (e.g., freshwater fish, amphibians, and waterfowl) and native terrestrial animals (rodents and shrews), as well as various invertebrates such as slugs, snails, crayfish, and insects [6,7]. This dietary flexibility allows the species to adapt to different ecosystems, and prey composition varies depending on regional characteristics and prey availability [8]. The broad dietary range and ecological adaptability of American mink position this species as a potential reservoir host for various infectious agents, including viruses, bacteria, fungi, protozoa, and helminths, which may be pathogenic to native wildlife species [5,9,10].

In Chile, several studies have documented the role of American mink as a carrier of important pathogens [11,12,13,14]. For instance, high prevalence rates of bacteria from the genus *Leptospira* spp. and *Mycobacterium bovis* have been detected in southern Chilean mink populations, confirming their potential role as suitable reservoir hosts of leptospirosis and tuberculosis in aquatic environments [11,12]. These infections typically occur when American minks either encounter contaminated freshwater bodies or consume infected rodents [13]. Such zoonotic diseases, which affect both animals and humans, demonstrate the capacity of this mustelid to serve as a reservoir for pathogens with significant public health implications [14]. In Cape Horn, American minks interact with other invasive species, such as beavers (*Castor canadiensis*, *Castor fiber*) and muskrats (*Ondata zibethicus*), facilitating pathogen transmission among species [15]. This interaction among invasive species not only threatens endemic biodiversity of Chile but also increases the disease burden within affected ecosystems [16]. Recently, *Trichinella spiralis*, the causative agent of trichinellosis—a zoonotic disease of public health importance worldwide—was recorded for the first time in South American mink populations [16]. The generalist diet of American minks facilitates the circulation of this zoonotic parasite in both wild and peridomestic cycles [16].

Cardiorespiratory nematode parasites in domestic- and wild carnivores have been extensively studied and documented worldwide, particularly in Europe, where various host species including domestic dogs, wolves (*Canis lupus*), badgers (*Meles meles*), raccoons (*Procyonis lotor*), red foxes (*Vulpes vulpes*), and American minks have been investigated [17,18,19,20]. Parasitic nematode species such as *Angiostrongylus daskalovi*, *Angiostrongylus vasorum*, *Crenosoma melesi*, *Crenosoma vulpis*, and *Aelurostrongylus* spp., all belonging to the superfamily Metastrongyloidea, have been reported in these hosts [21,22]. In a study conducted in Spain, a low prevalence of cardiorespiratory nematodes was reported in American mink, with only one individual testing positive for *Angiostrongylus daskalovi* [23]. Similarly, in Poland, metastrongyloid nematodes were detected in 3.8% of examined mink, with *Crenosoma* spp. being the most frequently identified genus [24]. These findings underscore the susceptibility of American mink to infection by cardiorespiratory nematodes and their potential role in the epidemiology of these neglected parasites.

The genus *Aelurostrongylus* comprises several species of metastrongyloid nematodes that primarily parasitize the pulmonary parenchyma and bronchioles of felids and other carnivores [21,25,26]. *A. abstrusus* is the most well-characterized species and is recognized as an important respiratory pathogen in domestic- and wild felids [e.g., wild cats (*Felis silvestris*), Eurasian lynxes (*Lynx lynx*)] worldwide [26,27,28]. The life cycle of *Aelurostrongylus* spp. is indirect and requires gastropod intermediate hosts (snails and slugs) for larval development [23,24,26]. Definitive hosts become infected by ingesting infected gastropods or paratenic hosts (such as rodents, birds, or amphibians) that have consumed infected gastropods [21,25,26,27,28,29,30,31,32]. Given the dietary habits of American mink, which include gastropods and small vertebrates that may serve as paratenic hosts, this species is potentially susceptible to *Aelurostrongylus* infections [8,33].

Despite the growing body of knowledge regarding parasitic infections in American mink populations in the Northern hemisphere, data about cardiorespiratory nematodes in South American mink populations remains scarce. To date, no studies have documented *Aelurostrongylus* spp. infection in American mink in Chile or elsewhere in South America. Understanding the parasitic fauna of invasive species is crucial for assessing their potential impact on native wildlife health and ecosystem dynamics such as the ones to be present in Chile [34]. Furthermore, identifying novel host–parasite associations contributes to our understanding of parasite transmission pathways and the ecological factors that facilitate pathogen spillover between invasive and native species [34,35].

The objective of this study was to investigate the presence of cardiorespiratory metastrongyloid nematode genera, with emphasis on *Aelurostrongylus*, *Angiostrongylus* and *Crenosoma*, in wild neozoan American mink populations from southern Chile. This research provides the first documentation of patent pulmonary *Aelurostrongylus* nematode infections in American minks in South America and contributes to the understanding of the parasitic ecology of this invasive species in fragile Chilean ecosystems with a variety of endemic wild carnivorous species including the native Chilean lesser grison (*Galictis cuja*), wild felids [e.g., Pampas cat (*Leopardus colocola*), guinia (*Leopardus guigna*) and wild canids [e.g., Andean fox (*Pseudalopex culpeus*), Darwin’s fox (*Lycalopex fulvipes*)] among others.

## 2. Materials and Methods

### 2.1. Study Area and Sample Collection

Between 2024 and 2025, a total of 27 American minks (*N. vison*) carcasses were collected from Los Lagos Region (41°28′ S, 72°56′ W). These regions are characterized by temperate rainforest ecosystems with abundant freshwater bodies, including rivers, lakes, and wetlands, which provide suitable habitat for American mink populations. The specimens were obtained through collaboration with The National Agricultural and Livestock Service [Servicio Agricola y Ganadero (SAG)] from Chile and local hunters as part of invasive species control programs. All carcasses were transported to the Institute of Pharmacology and Morphophysiology at Universidad Austral de Chile (UACh) in refrigerated conditions and processed within 24 h of collection.

### 2.2. Bronchioalveolar Lavage (BAL)

BAL samples were collected from all animals to investigate the presence of metastrongyloid pulmonary nematodes. BAL samples were carried out following a standard protocol. Animals were positioned in dorsal recumbency, and the ventral cervical region was clipped and aseptically prepared. A midline incision through the skin and underlying cervical musculature was made using a straight-blade scalpel to facilitate endotracheal intubation. Then, a 10 mL syringe with a cannula attached to the cap was introduced directly into the trachea through the distal airways until the tip of the canula lodged in the bronchial tree. Then, 8 mL of sterile saline solution (0.9% NaCl) was manually instilled via the syringe and immediately aspirated by manual aspiration. The aspirated aliquot of approx. 5 mL was stored in sterile plastic Falcon tubes and then cooled at 4 °C. Recovered BAL fluid was stored in sterile containers and centrifugated for later parasitological examination. BAL samples were centrifugated at 100 *g* for 5 min and the supernatant was removed. Examination included morphological assessment of parasites. The BAL fluid collected samples were placed in Petri dishes and examined under a stereomicroscope at 20× magnification to observe nematode larvae. The base of the dish was scanned for larvae, and mucus was examined at different planes of focus to detect trapped larvae. Samples of BAL fluid were recorded as either positive or negative for metastrongyloid larvae. Morphological and morphometrical features used to identify first-stage larvae (L1) of *A. abstrusus* were length of 360 to 400 um, lack of a sheath, a coiled or S-shaped appearance and a subterminal spine in the caudal end [36,37].

### 2.3. Necropsy and Parasitological Examination

Complete necropsies were performed on all specimens following standardized protocols. Particular attention was given to the cardiorespiratory system, including the trachea, bronchi, lungs, and heart. Organs were systematically examined for the presence of macroscopic metastrongyloid parasites. Lung tissue was carefully dissected, and the parenchyma was compressed between glass plates to facilitate the visualization of different parasite stages (i.e., eggs, larvae, adults) within the bronchioles and alveoli. Any visible preserved nematodes were collected using fine forceps and preserved in 70% ethanol for subsequent morphological and molecular analysis. Additionally, fecal samples were collected from the large intestine of each specimen and examined using the Baermann technique to detect L1 of metastrongyloid nematodes. Approximately 5 g of feces were placed in a funnel lined with gauze and suspended in tap water for 24 h. The sediment was then collected and examined microscopically at 100× and 400× magnification for the presence of larvae.

### 2.4. Morphological Identification

Recovered nematodes and larvae were examined under a light microscope (Olympus CX31, Tokyo, Japan) equipped with a digital camera. Morphological identification was based on characteristic features described in the taxonomic literature, including body size, shape, and the presence of specific morphological structures. For first-stage larvae, particular attention was paid to the morphology of the tail, including the presence and shape of the dorsal spine, which is a key diagnostic feature for differentiating metastrongyloid genera [24]. Measurements were taken using calibrated ocular micrometers, and photomicrographs were captured for documentation.

### 2.5. Tissue Sampling for Histological Examination

Lung tissue samples were obtained from four American minks and immediately fixed in 10% neutral buffered formalin. Following routine paraffin embedding, tissue sections were stained with hematoxylin and eosin (H&E) and evaluated by light microscopy for histopathological changes, according to previously established protocols.

### 2.6. Molecular Identification

To confirm the superfamily level molecular identity of the recovered nematodes, conventional PCR was performed on DNA extracted from one preserved specimen. Genomic DNA was extracted from nematodes stored in 70% ethanol using the E.Z.N.A.^®^ Tissue DNA Kit (Omega Bio-tek, Norcross, GA, USA), following the manufacturer’s instructions. A universal primer pair targeting a conserved region within the superfamily Metastrongyloidea was used: forward primer U1 (AaGp28Sa1): 5′-CGAGTRATATGTATGCCATT-3′ and reverse primer U2 (AaGp28Ss1): 5′-AGGCATAGTTCACCATCT-3′. PCR reactions were performed using GoTaq^®^ Green Master Mix 2X (Promega Corporation, Fitchburg, MA, USA) in a final volume of 25 μL, containing 1× Master Mix, 10 μM of each primer, and approximately 150 ng of template DNA, adjusted to volume with sterile nuclease-free water [38]. Thermal cycling conditions consisted of an initial denaturation at 94 °C for 5 min, followed by 35 cycles of denaturation at 94 °C for 30 s, annealing at 54 °C for 30 s, and extension at 72 °C for 30 s, with a final extension at 72 °C for 5 min. PCR products were resolved by electrophoresis on 2% agarose gels (Fermelo Biotec, Santiago, Chile) prepared in TBE (Tris-Borate-EDTA) buffer and supplemented with SafeView Plus intercalating stain (Fermelo Biotec, Santiago, Chile). Electrophoresis was carried out at 110 V for 40 min, and gels were visualized under UV light.

### 2.7. Ethical Considerations

All procedures involving animal specimens were conducted in accordance with Chilean legislation regarding wildlife research and invasive species management. The study was formally reviewed by the Institutional Committee for Animal Care and Use (Comité Institucional para el Cuidado y Uso de Animales, CICUA) of the Universidad Austral de Chile, which determined that the research was exempt from ethical authorization, as it did not involve experimental procedures or interventions on live animals. All specimens consisted exclusively of carcasses of American minks provided by the Chilean Agricultural and Livestock Service (Servicio Agrícola y Ganadero, SAG), obtained within the framework of official invasive exotic species control programs, pursuant to Article 6 of the Hunting Law Regulation (D.S. N°05, 1998). The research team had no participation in the capture, handling, euthanasia, or killing of the animals, and was solely involved in the collection and analysis of biological samples from the carcasses.

Artificial Intelligence tool (SciSpace AI, Typeset Inc., accessed May 2026) was used for assisting in grammar and manuscript organization.

## 3. Results

Among the 27 individuals captured and analyzed, 85.2% were males and 14.8% were females. The majority of specimens were classified as adults (74%), while juveniles accounted for 26%. Regarding temporal distribution, 91.3% of the captures were concentrated in September 2024, with the 10th being the date with the highest number of captures (34.8%). Geographically, all individuals came from nine areas at the Los Lagos Region, with San Pablo being the most represented with 30.4% (7/23) of the records, followed by Puerto Montt with 17.3% (4/23) and Puerto Varas with 13% (3/23).

Of the total animals analyzed, 9 tested positive for the presence of metastrongyloid parasitic stages (Cases 1; 3; 6; 8; 9; 10; 15; 16 and 17), corresponding to 33.3%. All of these positive cases were diagnosed using the BAL technique and coincided with positive results in the other tests, including the Baermann technique and histopathological analysis (Table 1).

### 3.1. BAL and Morphological Findings

L1 larvae with curvy caudal end suggestive of patent *Aelurostrongylus* infections were recovered from 9 specimens. These larvae had a length dimension of 267–332 µm (Table 2, Figure 1). Collected metastrongyloid L1 found at the Baermann examination were as well identified based on morphometric and morphological features (i.e., pointed cephalic end, S-shaped tail with knob-like or small finger-like projections at tip of cuticular spines) (Figure 1).

L1 stage larvae were observed on BAL in 9 individuals, with approximately 300 µm long. They had a thin, smooth cuticle with no visible external ornamentation. The anterior end was rounded, lacking sharp structures or distinguishable protrusions, and no defined oral structures were evident. The posterior end was sharp, ending in a point with a deep notch at the caudal end. Neither visible accessory bifurcations nor projections were observed (Figure 1). Two eggs were observed by BAL in case 1, with approximate dimensions of 87 µm and 63 µm in length, respectively. Both eggs were ellipsoidal in shape and had thin egg shells (Figure 2). The morphological features and dimension of the L1 and pre-adult larvae and the anatomic locations of the nematodes were indicative of *Aelurostrongylus* spp. [39].

Positive cases came from Puerto Montt (3 cases) and the rural areas of Purranque (2 cases), San Pablo (2 cases), Fresia (1 case), and Puyehue (1 case) (Figure 3). Eight infected specimens were adults and only one juvenile (Table 1).

### 3.2. Histopathological Analysis

None of the infected American minks showed obvious macroscopic lesions in the respiratory tract during necropsy. The lungs appeared grossly normal, with no evidence of consolidation, nodules, or hemorrhage. All metastrongylod-infected individuals were in good body condition, suggesting that these infections were of low intensity and did not cause significant clinical disease at the time of death. Histopathological analysis was performed on a total of 9 individuals (55.5%). All individuals with the presence of parasitic stages in the lung parenchyma had larvae in BAL and Baermann techniques (refer to Table 1). On histological examination, pre-adult larvae were found in pulmonary parenchymal tissue (Figure 4) on 5 specimens of American minks. Nematodes appereared to induce small inflamatory reaction other than a slight increase in cellularity of the adjacent to terminal bronchial airways and alvealoar walls. Histopathological examination of specimen 3 revealed multiple adult gravid female parasitic nematodes in the airway lumen, most with multiple internal structures consistent with uteri filled with embryonated eggs containing larvae. In addition, abundant diffuse alveolar fluid was observed with few macrophages in the lumen and generalized congestion. Positive case 8 revealed a moderate presence of parasitic nematodes in the airway lumen, with the presence of embryonated eggs inside the uteri. Generalized congestion and fluid in the alveolar lumen were also observed. Positive case 9 revealed abundant adult nematodes in the airway lumen, most with multiple internal structures consistent with thin-shelled eggs containing larvae, and few free larvae were also observed. Alveolar fluid was also present in former case. In case 10, abundant adult nematodes were observed in the airway lumen, with multiple internal structures compatible with embryonated eggs. Some free-roaming larvae were also detected (please refer to Figure 4C). Although the presence of homogeneous proteaceous material in the pulmonary parenchyma (fluid) is usually interpreted as alveolar edema, the absence of macrophages in this series of cases could suggest a *post-mortem* change.

### 3.3. Molecular Analysis

Conventional PCR targeting a conserved region of the superfamily Metastrongyloidea was performed on one positive specimen (case 17). Amplification with the universal primer pair U1/U2 yielded a specific band of the expected size, confirming the presence of a nematode belonging to the superfamily Metastrongyloidea. This result is consistent with, and provides molecular support for, the morphological identification of the parasites as *Aelurostrongylus* spp. obtained through BAL and histopathological analyses. No amplification product was detected in the negative control. Molecular analysis was not performed on the remaining positive specimens due to limitations in available preserved material suitable for DNA extraction.

## 4. Discussion

This study reports the first detection of *Aelurostrongylus* spp. infections in invasive American minks (*N. vison*) in South America, specifically in Southern Chile. The morphological and morphometric analysis confirmed the presence of parasites closely related to *A. abstrusus*, a metastrongyloid nematode primarily known as a respiratory parasite of domestic- and wild felids [26,35]. This finding expands the known host range of the genus *Aelurostrongylus* and highlights the potential role of invasive American minks as natural reservoir hosts for cardiorespiratory parasites in South American ecosystems.

The study prevalence of *Aelurostrongylus* infection observed in this study (33.3%) is higher to prevalence rates reported for other metastrongyloid nematodes in American mink populations in Europe. For example, studies in Poland reported a 3.8% prevalence of metastrongyloid infections in mink, primarily involving *Crenosoma* spp. [24]. In Spain, a low prevalence of *A. daskalovi*, a parasite also occurring in badgers, was documented in American mink [23]. A study conducted in Spain reported a low prevalence of cardiorespiratory nematodes in the American mink, with only one individual testing positive for *A. abstrusus* out of 123 animals examined [40,41]. In a preceding study, also from Spain, *Aelurostrongylus pridhami* was identified in specimens of European mink (*Mustela lutreola*) [42].

A higher frequency of cardiorespiratory parasites was observed in male (85.2%) and adult (74%) mink, which could be related to demographic factors of the sampled population, behavioral differences, or greater exposure to the capture methods used. This pattern is consistent with findings reported in other wild carnivore populations, where males and adults consistently exhibit greater infection rates [43]. Positivity was also considerably higher in adults (29.63%) than in juveniles (3.7%), which could reflect greater cumulative exposure to the infectious agent, greater mobility, or predatory experience that favors contact with intermediate hosts [26,44]. However, this difference could be related to ecological and behavioral aspects and not necessarily indicate a sex predisposition. In mustelids, such as the American mink, males exhibit greater territorial mobility, wider home ranges, and a diet that includes larger prey compared to females [45]. Intraspecific resource competition and spacing dynamics further reinforce these sex-based differences in spatial behavior and habitat use [46]. These differences may increase exposure to infected hosts and, therefore, the risk of infection. Furthermore, sexual dimorphism and ecological niche separation have been documented as factors influencing infection dynamics in wild carnivores, reinforcing this hypothesis [47].

These relatively low prevalence rates may reflect the opportunistic nature of these infections and the dependence on specific ecological conditions that facilitate transmission, including the availability of suitable intermediate and paratenic hosts.

The transmission ecology of *Aelurostrongylus* spp. involves complex life cycles requiring obligate gastropod intermediate hosts (snails, semi-slugs and slugs) for larval development [28,31,36,48,49]. Definitive hosts become infected through the ingestion of either infected gastropods or paratenic hosts such as rodents, birds, reptiles, and amphibians that have consumed infected gastropods carrying L3 [21,28,35,36,37]. The dietary habits of American minks in Chile, which include a variety of endemic occurring aquatic and terrestrial prey species, provide multiple potential routes of exposure to *Aelurostrongylus* infections [6,8]. Minks are well-known to consume gastropods, particularly in aquatic environments where these invertebrates are abundant [6,8]. Additionally, mink frequently prey on small mammals and amphibians that may serve as paratenic hosts, further increasing the risk of parasite transmission [8].

The detection of patent aelurostrongylosis in American minks raises important questions about the source of infection and the potential for parasite spillover between invasive and native species. In Chile, several native carnivore species, including the kodkod (*L. guigna*), the Pampas cat (*L. colocola*) and the Geoffroy’s cat (*Leopardus geoffroyi*), share habitats with American minks and could potentially serve as reservoir hosts for *Aelurostrongylus* spp. [50]. Alternatively, American minks may have acquired the infection from domestic- and wild cats, which are known to harbor *A. abstrusus* and being present in rural areas throughout Southern Chile [32,50,51,52]. Further studies investigating the prevalence of *Aelurostrongylus* infection in native and domestic carnivores in this region are needed to elucidate transmission dynamics and assess the potential for bidirectional pathogen exchange between invasive and protected native wild felids, reptiles, amphibians and rodent species acting as paratenic hosts. The same hold true for the possible negative impact of this infection on endemic gastropod fauna within protected temperate rainfall forests of Southern Chile.

This finding is consistent with the hypothesis that genus *Aelurostrongylus* may have been introduced to Chile through infected domestic cats and/or other carnivores, rather than representing a native parasite species. However, definitive species identification would require morphological examination of adult parasites, which were not recovered in this study. The use of additional molecular markers, such as the internal transcribed spacer 2 (ITS-2) region, could provide further resolution for species-level identification and phylogeographic analysis [53].

From a pathological perspective, *Aelurostrongylus* infections in felids can cause a range of clinical signs, from subclinical infections to severe respiratory disease characterized by coughing, dyspnea, and exercise intolerance [48,49]. The severity of clinical signs typically depends on the intensity of infection and the immune status of the host [39,40]. In this study, none of the *Aelurostrongylus*-infected minks exhibited obvious macroscopic lung lesions, suggesting that the infections were of low intensity. However, the absence of histopathological examination limits our ability to assess the presence of microscopic lesions or inflammatory responses. Future studies should include detailed histopathological analysis to better characterize the pathological impact of patent *Aelurostrongylus* infections not only in invasive American minks but also in Chilean lesser grison (*G. cuja*) and endemic gastropod fauna acting as obligate intermediate hosts.

The ecological implications of *Aelurostrongylus* infection in invasive American mink populations warrant consideration. As an invasive species and stated above, American minks can alter ecosystem dynamics through predation on native fauna and competition with native carnivores [2,51]. The introduction or amplification of parasites by invasive species can further impact native wildlife populations through pathogen spillover, a phenomenon that has been documented for various invasive species worldwide [31,33,51]. In the case of *Aelurostrongylus* spp., the potential for transmission to native endangered wild felids or other susceptible carnivores could pose health risks to already threatened populations. For example, the kodkod (guiña), a small native felid endemic to Southern Chile and Argentina, is classified as vulnerable by the International Union for Conservation of Nature (IUCN) and could be particularly susceptible to novel parasitic infections [52].

The role of American mink as a reservoir host for patent *Aelurostrongylus* spp. may be influenced by several ecological factors. The semiaquatic lifestyle of mink and their preference for riparian habitats place them in close proximity to gastropod populations, which are particularly abundant in moist environments [29]. Additionally, the generalist diet of minks increases the likelihood of consuming infected intermediate or paratenic hosts [6,8]. Climate and environmental conditions in Southern Chile, characterized by high rainfall and moderate temperatures, provide favorable conditions for gastropod populations and may facilitate the transmission of metastrongyloid parasites [29].

This study has several limitations that should be acknowledged. First, the sample size was relatively small (*n* = 27), and specimens were collected opportunistically rather than through systematic sampling. This may have introduced sampling bias and limited our ability to accurately estimate prevalence and identify risk factors associated with infection. Second, adult parasites were not recovered during necropsy, precluding detailed morphological characterization and definitive species identification based on adult morphology. Third, the study was conducted in a limited geographical area (i.e., Los Ríos and Los Lagos regions), and the findings may not be representative of American mink populations in other parts of Chile or South America. Finally, we did not investigate the prevalence of *Aelurostrongylus* infection in potential intermediate hosts (gastropods) or paratenic hosts (rodents, amphibians) in the study area, which would provide valuable information about transmission dynamics but national permission issues for sampling protected Chilean endemic fauna are to be considered.

Despite these limitations, this study provides important baseline data on metastrongyloid parasites in neozoan American minks in South America and highlights the need for continued surveillance of lungworm parasitic infections in invasive carnivore populations. Future research should aim to: (1) conduct larger-scale surveys to better estimate the prevalence and geographical distribution of aelurostrongylosis infection in American minks across Chile as well as closely related Chilean lesson grison populations; (2) investigate the prevalence of *Aelurostrongylus* spp. in native- and domestic felids to assess the potential for parasite spillover; (3) characterize the intermediate- and paratenic host communities involved in parasite transmission; (4) perform detailed histopathological studies to assess the pathological impact of mustelid aelurostrongylosis; and (5) use additional molecular markers to clarify the taxonomic identity and/or phylogeographic relationships of *Aelurostrongylus* populations in South America.

## 5. Conclusions

This study provides the first evidence of patent *Aelurostrongylus* spp. infections in American minks (*N. vison*) in South America, specifically in Southern Chile. The detection of this parasite genus in an invasive mustelid species highlights the potential role of American mink as reservoir host for cardiorespiratory parasites and raises concerns about possible pathogen spillover to native carnivore populations. These findings underscore the importance of monitoring parasitic infections in invasive species as part of comprehensive wildlife health surveillance programs for biodiversity preservation. Thus, further research is needed to elucidate the transmission dynamics, host range, and ecological impact of *Aelurostrongylus* spp. in South American endemic and fragile ecosystems and to assess the potential risks to domestic animals and native wildlife.

## Figures and Tables

**Figure 1:**
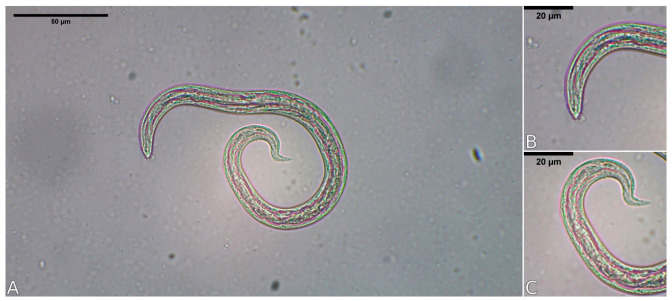
*Aelurostrongylus* spp. larvae recovered from bronchioalveolar lavage from culled American minks (*Neogale vison*). 40× view of a larva obtained using the Baermann technique in mink Nº3 in this study (**A**). 40× view of a larva showing the craneal end (**B**). 40 view of the parasite larva showing the caudal end (**C**).

**Figure 2:**
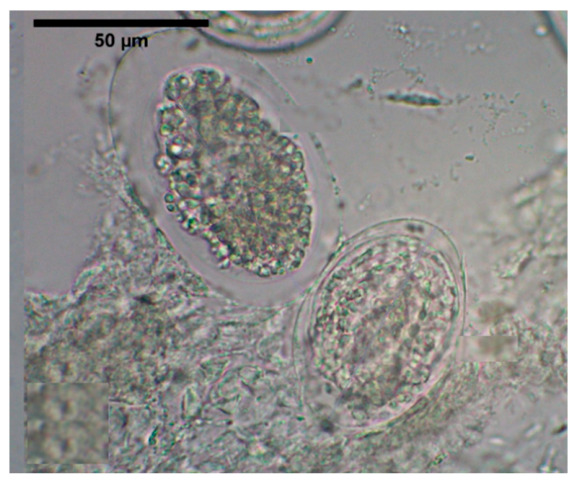
40× view of embryonated thin-shelled eggs containing a morula and a larval stage recovered by bronchoalveolar lavage (BAL) from one American mink (*Neogale vison*).

**Figure 3:**
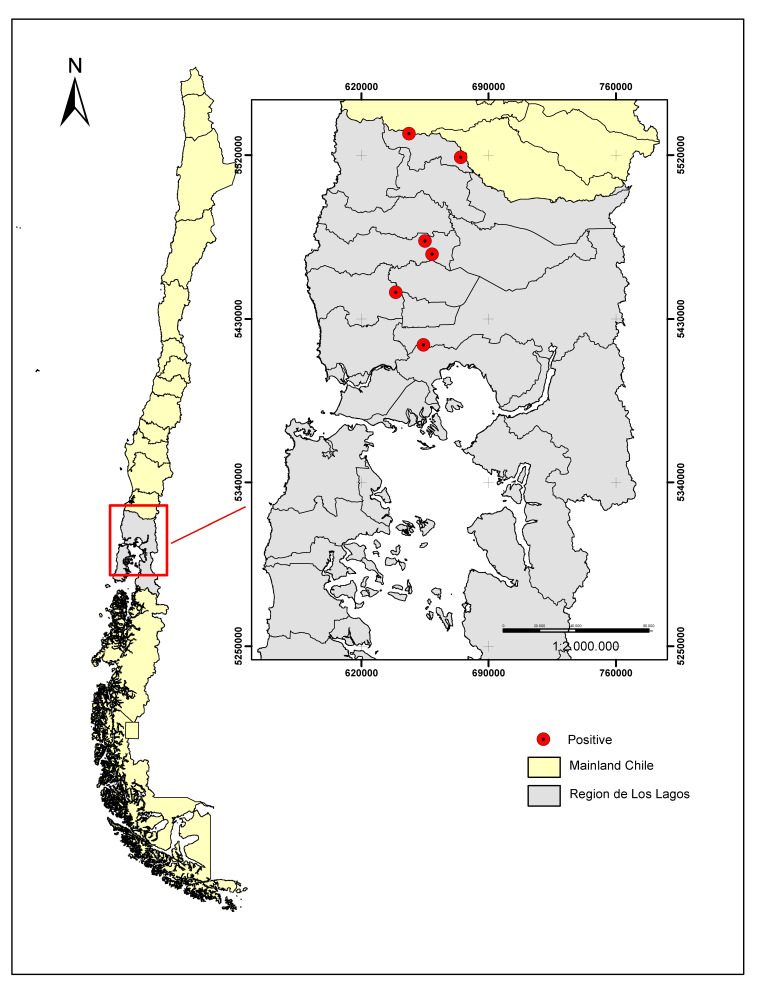
Georeferenced map of Southern Chile showing the geographic location of positive American minks (*Neogale vision*) for the presence of metastrongyloid cardiopulmonary nematodes.

**Figure 4:**
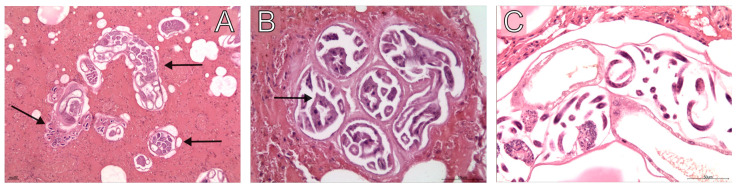
Histopathological images of American mink (*Neogale vision*) lung tissue with the presence of *Aelurostrongylus* spp. nematodes. (**A**) Cross-section of the lung of an American mink (number 9, 10×). The black arrows indicate the presence of adult parasites cross-sections located in the airways. (**B**) Cross-sections of *Aelurostrongylus* nematodes at the lung parenchyma of case number 8, 40×. The black arrow indicates structures compatible with the intestine of an adult nematode. (**C**) Histological image of the lung parenchyma of case number 10, 40×. Larvae and embryonated thin-shelled eggs are observed inside the uterus of a gravid *Aelustrongylus* nematode.

**Table 1:** Demographic features and diagnosis of pulmonary parasites of 27 invasive American mink (*Neogale vison*) specimens from the Los Lagos Region, Southern Chile.

		N° Positives/N° of Total Tested	%
Test	BAL ^1^ (L1 larvae)	9/27	33.33
	Baermann (L1 larvae)	4/27	14.81
	BAL ^1^ (eggs)	1/27	3.7
	Histopathology	5/9	55.55
Sex	Males	9/27	33.33
	Females	0/27	0
Location	Purranque	2/23	8.7
	Fresia	1/23	4.35
	Puyehue	1/23	4.35
	Puerto Montt	3/23	13.04
	San Pablo	2/23	8.7
Age	Adult	8/27	29.63
	Juvenile	1/27	3.7

^1^ BAL: bronchoalveolar lavage.

**Table 2:** Metastrongyloid first-stage larvae (L1) measurements obtained from 9 American minks (*Neogale vison*).

L1 Larvae Length (µm)		
Specimen	BAL	Baermann Examination
1	321.2	ND
3	267.8	324.5
6	283.1	291.9
8	295.8	Negative
9	289.4	281.9
10	332.8	Negative
15	283.4	269.5
16	278.6	Negative
17	304.6	Negative

BAL: Bronchoalveolar lavage; ND: non data.

## Data Availability

The original contributions presented in this study are included in the article. Further inquiries can be directed to the corresponding author.
